# *N*′-[5-(4-Nitro­phen­yl)furan-2-yl­methyl­idene]-*N*,*N*-diphenyl­hydrazine

**DOI:** 10.1107/S1600536810027388

**Published:** 2010-07-17

**Authors:** Angel Mendoza, Blanca M. Cabrera-Vivas, Ruth Meléndrez-Luevano, Teresa Pacheco-Álvarez, Vladimir Carranza

**Affiliations:** a Centro de Química, ICUAP, Benemérita Universidad Autónoma de Puebla, Puebla, Pue, Mexico; b Facultad de Ciencias Químicas, Benemérita Universidad Autónoma de Puebla, Puebla, Pue, Mexico

## Abstract

The title compound, C_23_H_17_N_3_O_3_, has an *E* configuration with respect to the C=N bond. The dihedral angle between the two phenyl rings bonded to the hydrazine group is 86.45 (13)°. The furan ring makes dihedral angles of 3.4 (2) and 7.06 (13)°, respectively, with the methyl­idenehydrazine C=N—N plane and the benzene ring.

## Related literature

For applications of hydrazones, see: Kobotayeva *et al.* (2001 ▶); Barlow *et al.* (2000 ▶); Knight *et al.* (2000 ▶); Ros *et al.* (2008 ▶). For related structures, see: Clulow *et al.* (2008 ▶); Motherwell & Ramsay (2007 ▶). For bond-length data, see: Allen *et al.* (1987 ▶). 
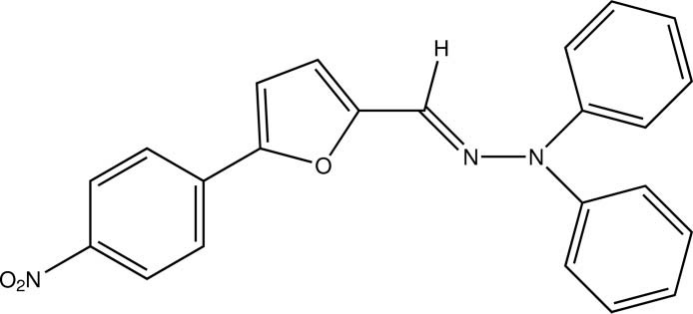


## Experimental

### 

#### Crystal data

C_23_H_17_N_3_O_3_
*M* *_r_* = 383.4
Monoclinic, 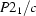

*a* = 16.815 (3) Å
*b* = 8.602 (1) Å
*c* = 13.340 (2) Å
β = 95.64 (2)°
*V* = 1920.2 (6) Å^3^
*Z* = 4
Mo *K*α radiation
μ = 0.09 mm^−1^
*T* = 293 K
0.4 × 0.4 × 0.15 mm

#### Data collection

Bruker P4 diffractometer
6417 measured reflections
5099 independent reflections
1970 reflections with *I* > 2σ(*I*)
*R*_int_ = 0.067
3 standard reflections every 97 reflections  intensity decay: 6%

#### Refinement

*R*[*F* ^2^ > 2σ(*F* ^2^)] = 0.058
*wR*(*F* ^2^) = 0.181
*S* = 0.97
5099 reflections
262 parameters
H-atom parameters constrained
Δρ_max_ = 0.18 e Å^−3^
Δρ_min_ = −0.20 e Å^−3^

### 

Data collection: *XSCANS* (Siemens, 1994 ▶); cell refinement: *XSCANS*; data reduction: *XSCANS*; program(s) used to solve structure: *SIR2004* (Burla *et al.*, 2005 ▶); program(s) used to refine structure: *SHELXL97* (Sheldrick, 2008 ▶); molecular graphics: *ORTEP-3 for Windows* (Farrugia, 1997 ▶); software used to prepare material for publication: *WinGX* (Farrugia, 1999 ▶).

## Supplementary Material

Crystal structure: contains datablocks global, I. DOI: 10.1107/S1600536810027388/is2572sup1.cif

Structure factors: contains datablocks I. DOI: 10.1107/S1600536810027388/is2572Isup2.hkl

Additional supplementary materials:  crystallographic information; 3D view; checkCIF report

## Figures and Tables

**Fig. 1.**
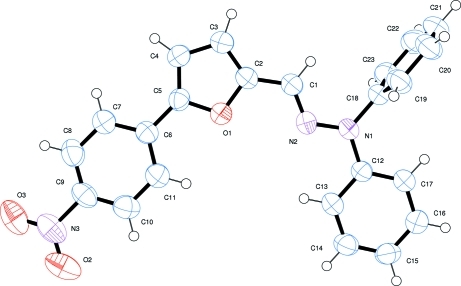
The molecular structure of compound I, with atom labels and 50% probability displacement ellipsoids for non-H atoms.

**Fig. 2.**
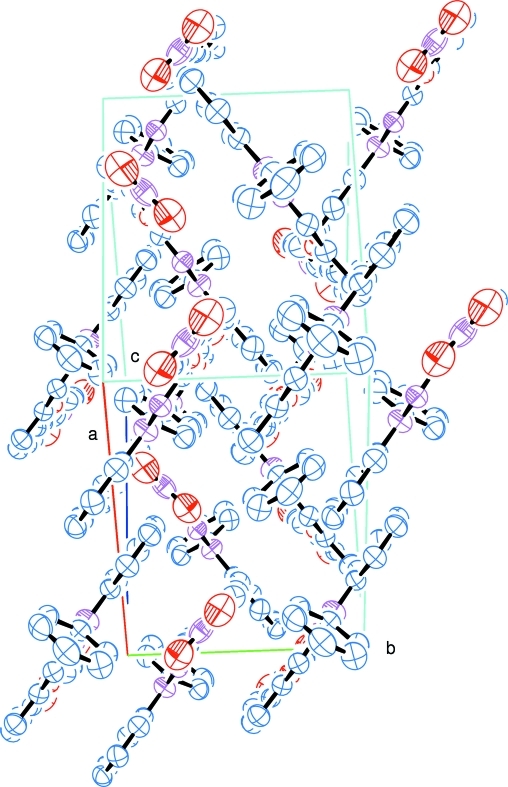
Molecular packing of compound I showing cross-linked paking.

## supplementary crystallographic information

### Comment

Several hydrazones, including diphenylhydrazones, can be used as hole carriers in thin film organic photoconductors applied to electrographic processes in printers and photocopiers, plasticizers, polymer stabilizers, antioxidants and polymer initiators (Kobotayeva *et al.*, 2001; Barlow *et al.*, 2000; Knight *et al.*, 2000). Hydroxylated hydrazones are used as herbicides, insecticides and growth promoters in plants due to their biological activity (Ros *et al.*, 2008).

The title compound, I, presents an *E* configuration with *N*,*N*-diphenyl group opposite to 5-(4-nitrophenyl) furane group from the N2=C1 double bond. The asymmetric unit of compound I shows a non-planar structure for a phenyl ring next to N—N group, with a torsion angle N2—N1—C18—C23 = 89.1 (3)°, which is similar to some related structures previously reported (Motherwell & Ramsay, 2007). The N—N distance [1.356 (3) Å] is shorter than found in free diphenylhydrazine [1.418 (2) Å] (Clulow *et al.*, 2008) and similar to related structure with 2,4 dinitrophenyl hydrazone group [1.383 (4) Å] (Motherwell & Ramsay, 2007). Nitrophenyl ring present a torsion angle of 6.7 (3)° from the furane ring. The N2=C1 double bond distance [1.286 (3) Å] is longer than the N=C typical bond distance (Allen *et al.*, 1987), probably due to π conjugation along all the molecule. The crystal packing shows van der Waals interactions, one of them between O1···H—C4 (2.62 Å) parallel to the [111] base vector (symmetry, -*x* + 2, *y* - 1/2, -*z* + 3/2), and two more interactions O2···H—C14 (2.66 Å) and O2···H—C19 (2.69 Å) parallel to the [1–11] base vector with symmetry operators -*x* + 2, *y* + 1/2, -*z* + 1/2 and -*x* + 2, -*y* + 1, -*z* + 1, respectively. These interactions are building up a cross-linked packing (Fig. 2).

### Experimental

*N*,*N*-diphenylhydrazine (0.731 mg, 3.31 mmol) was dissolved in ethanol, then acetic acid (0.5 ml) was added slowly into this solution while stirring. 5-(4-Nitrophenyl)furan-2-carbaldehyde (600 mg, 2.76 mmol) was added drop by drop into the above solution with strong stirring and the resulting mixture was kept at atmospheric temperature until it became orange-red solution. After one hour the orange-red solution turns to be precipitated. The mixture was separated with filtration in vacuum system and the precipitate was washed three times with methanol. Recrystallization was performed three times with acetonitrile to obtain suitable red crystals for X-ray analysis. Yield: 860 mg (78%) at 25 °C, m.p. 166–168 °C. FT IR (film): (cm^-1^): 3119 ν(C—H), 1683, 1600 ν(C=N), 1667–2000 ν(Ph), 1513 ν(Ph—NO_2_), 1221 ν(=C—O), 851 ν(Ph—NO_2_). EI—MS: m/*z* 383 *M*^+^.

### Refinement

H atoms linked to C atoms were placed in geometrical idealized positions and refined as riding on their parent atoms, with C—H = 0.93 Å and with *U*_iso_(H) = 1.2*U*_eq_(C).

### Crystal data

**Table:**

C_23_H_17_N_3_O_3_	*F*(000) = 800
*M**_r_* = 383.4	*D*_x_ = 1.326 Mg m^−^^3^
Monoclinic, *P*2_1_/*c*	Mo *K*α radiation, λ = 0.71073 Å
*a* = 16.815 (3) Å	Cell parameters from 48 reflections
*b* = 8.602 (1) Å	θ = 4.9–24.8°
*c* = 13.340 (2) Å	µ = 0.09 mm^−^^1^
β = 95.64 (2)°	*T* = 293 K
*V* = 1920.2 (6) Å^3^	Prism, red
*Z* = 4	0.4 × 0.4 × 0.15 mm

### Data collection

**Table:**

Bruker P4 diffractometer	*R*_int_ = 0.067
Radiation source: fine-focus sealed tube	θ_max_ = 29°, θ_min_ = 2.4°
graphite	*h* = −22→22
2θ/ω scans	*k* = −11→1
6417 measured reflections	*l* = −1→18
5099 independent reflections	3 standard reflections every 97 reflections
1970 reflections with *I* > 2σ(*I*)	intensity decay: 6%

### Refinement

**Table:**

Refinement on *F*^2^	Primary atom site location: structure-invariant direct methods
Least-squares matrix: full	Secondary atom site location: difference Fourier map
*R*[*F*^2^ > 2σ(*F*^2^)] = 0.058	Hydrogen site location: inferred from neighbouring sites
*wR*(*F*^2^) = 0.181	H-atom parameters constrained
*S* = 0.97	*w* = 1/[σ^2^(*F*_o_^2^) + (0.0736*P*)^2^] where *P* = (*F*_o_^2^ + 2*F*_c_^2^)/3
5099 reflections	(Δ/σ)_max_ < 0.001
262 parameters	Δρ_max_ = 0.18 e Å^−^^3^
0 restraints	Δρ_min_ = −0.20 e Å^−^^3^

### Special details

**Table:**

Geometry. All s.u.'s (except the s.u. in the dihedral angle between two l.s. planes) are estimated using the full covariance matrix. The cell s.u.'s are taken into account individually in the estimation of s.u.'s in distances, angles and torsion angles; correlations between s.u.'s in cell parameters are only used when they are defined by crystal symmetry. An approximate (isotropic) treatment of cell s.u.'s is used for estimating s.u.'s involving l.s. planes.
Refinement. Refinement of *F*^2^ against ALL reflections. The weighted *R*-factor *wR* and goodness of fit *S* are based on *F*^2^, conventional *R*-factors *R* are based on *F*, with *F* set to zero for negative *F*^2^. The threshold expression of *F*^2^ > 2σ(*F*^2^) is used only for calculating *R*-factors(gt) *etc*. and is not relevant to the choice of reflections for refinement. *R*-factors based on *F*^2^ are statistically about twice as large as those based on *F*, and *R*- factors based on ALL data will be even larger.

### Fractional atomic coordinates and isotropic or equivalent isotropic displacement parameters (Å^2^)

**Table:**

	*x*	*y*	*z*	*U*_iso_*/*U*_eq_	
O1	0.93074 (9)	0.17913 (18)	0.63960 (10)	0.0517 (4)	
N2	0.78159 (11)	0.2996 (2)	0.63257 (14)	0.0538 (5)	
C5	1.00087 (13)	0.0981 (3)	0.66001 (16)	0.0493 (6)	
C6	1.05966 (13)	0.1186 (3)	0.58891 (17)	0.0501 (6)	
C12	0.68625 (13)	0.4575 (3)	0.54061 (16)	0.0497 (6)	
N1	0.70726 (11)	0.3624 (2)	0.62469 (14)	0.0570 (5)	
N3	1.23249 (16)	0.1772 (3)	0.38172 (19)	0.0764 (7)	
C1	0.80526 (14)	0.2131 (3)	0.70836 (17)	0.0525 (6)	
H1	0.772	0.1957	0.759	0.063*	
C9	1.17208 (15)	0.1553 (3)	0.45334 (18)	0.0610 (7)	
C17	0.61353 (14)	0.5339 (3)	0.53010 (17)	0.0588 (7)	
H17	0.5784	0.523	0.5793	0.071*	
C18	0.65427 (13)	0.3410 (3)	0.70192 (17)	0.0511 (6)	
C2	0.88302 (13)	0.1437 (3)	0.71450 (17)	0.0512 (6)	
C13	0.73808 (15)	0.4766 (3)	0.46598 (17)	0.0586 (6)	
H13	0.7872	0.426	0.4713	0.07*	
O2	1.21847 (15)	0.2726 (3)	0.31372 (17)	0.0991 (8)	
C7	1.12859 (15)	0.0287 (3)	0.59551 (19)	0.0657 (7)	
H7	1.137	−0.0447	0.6467	0.079*	
C15	0.64403 (16)	0.6445 (3)	0.37354 (18)	0.0629 (7)	
H15	0.6299	0.706	0.3173	0.076*	
O3	1.29323 (14)	0.1001 (3)	0.39341 (17)	0.1011 (8)	
C4	0.99722 (14)	0.0144 (3)	0.74533 (18)	0.0578 (7)	
H4	1.0369	−0.0506	0.7754	0.069*	
C14	0.71625 (16)	0.5701 (3)	0.38482 (18)	0.0644 (7)	
H14	0.7515	0.5835	0.336	0.077*	
C16	0.59273 (15)	0.6266 (3)	0.44678 (19)	0.0629 (7)	
H16	0.5436	0.6773	0.4403	0.075*	
C11	1.04867 (16)	0.2261 (3)	0.51146 (18)	0.0645 (7)	
H11	1.0027	0.2867	0.5052	0.077*	
C10	1.10469 (18)	0.2449 (3)	0.44350 (19)	0.0708 (8)	
H10	1.0967	0.3175	0.3918	0.085*	
C3	0.92220 (14)	0.0439 (3)	0.78013 (18)	0.0597 (7)	
H3	0.9032	0.0023	0.8376	0.072*	
C23	0.60325 (16)	0.2166 (3)	0.6983 (2)	0.0636 (7)	
H23	0.6024	0.1459	0.6455	0.076*	
C22	0.55305 (16)	0.1962 (4)	0.7731 (2)	0.0742 (8)	
H22	0.5183	0.1118	0.7707	0.089*	
C20	0.60633 (17)	0.4242 (4)	0.8553 (2)	0.0742 (8)	
H20	0.6078	0.494	0.9087	0.089*	
C8	1.18501 (15)	0.0460 (4)	0.5276 (2)	0.0694 (7)	
H8	1.2307	−0.0154	0.5323	0.083*	
C19	0.65619 (15)	0.4447 (3)	0.78046 (18)	0.0626 (7)	
H19	0.6911	0.5287	0.7831	0.075*	
C21	0.55436 (17)	0.3006 (4)	0.8511 (2)	0.0774 (9)	
H21	0.5201	0.2876	0.9011	0.093*	

### Atomic displacement parameters (Å^2^)

**Table:**

	*U*^11^	*U*^22^	*U*^33^	*U*^12^	*U*^13^	*U*^23^
O1	0.0515 (9)	0.0541 (10)	0.0499 (9)	0.0036 (8)	0.0077 (7)	0.0032 (8)
N2	0.0509 (12)	0.0572 (12)	0.0548 (12)	0.0066 (10)	0.0122 (9)	0.0004 (10)
C5	0.0488 (13)	0.0509 (14)	0.0478 (13)	0.0056 (11)	0.0033 (11)	−0.0031 (11)
C6	0.0510 (13)	0.0535 (15)	0.0459 (13)	−0.0033 (12)	0.0051 (10)	−0.0040 (12)
C12	0.0532 (14)	0.0521 (14)	0.0448 (12)	0.0028 (12)	0.0095 (10)	0.0012 (11)
N1	0.0516 (12)	0.0699 (14)	0.0521 (12)	0.0095 (11)	0.0176 (9)	0.0104 (11)
N3	0.0814 (18)	0.0832 (19)	0.0685 (16)	−0.0238 (16)	0.0271 (14)	−0.0264 (14)
C1	0.0529 (15)	0.0547 (14)	0.0512 (14)	0.0012 (12)	0.0118 (11)	0.0027 (12)
C9	0.0623 (16)	0.0718 (18)	0.0507 (14)	−0.0152 (14)	0.0146 (12)	−0.0119 (14)
C17	0.0580 (15)	0.0658 (17)	0.0546 (14)	0.0075 (13)	0.0152 (11)	0.0059 (13)
C18	0.0500 (13)	0.0559 (15)	0.0490 (13)	0.0054 (12)	0.0134 (11)	0.0083 (12)
C2	0.0530 (14)	0.0534 (14)	0.0484 (13)	−0.0013 (12)	0.0109 (11)	−0.0007 (12)
C13	0.0570 (14)	0.0664 (16)	0.0546 (14)	0.0056 (13)	0.0167 (11)	0.0031 (13)
O2	0.123 (2)	0.1055 (18)	0.0745 (13)	−0.0286 (15)	0.0401 (13)	−0.0019 (14)
C7	0.0621 (16)	0.0748 (18)	0.0616 (15)	0.0094 (14)	0.0135 (13)	0.0059 (14)
C15	0.0786 (19)	0.0553 (16)	0.0551 (15)	0.0026 (14)	0.0074 (13)	0.0089 (13)
O3	0.0833 (15)	0.117 (2)	0.1109 (17)	−0.0053 (15)	0.0475 (13)	−0.0214 (15)
C4	0.0601 (16)	0.0603 (16)	0.0531 (14)	0.0098 (13)	0.0060 (11)	0.0073 (13)
C14	0.0769 (18)	0.0642 (17)	0.0547 (15)	0.0022 (15)	0.0199 (13)	0.0056 (14)
C16	0.0627 (16)	0.0632 (17)	0.0633 (16)	0.0091 (13)	0.0092 (13)	0.0041 (14)
C11	0.0609 (17)	0.0750 (18)	0.0584 (15)	0.0047 (14)	0.0098 (13)	0.0042 (15)
C10	0.075 (2)	0.080 (2)	0.0583 (16)	−0.0018 (16)	0.0121 (14)	0.0087 (15)
C3	0.0635 (16)	0.0640 (16)	0.0530 (14)	0.0060 (14)	0.0125 (12)	0.0076 (13)
C23	0.0609 (16)	0.0618 (16)	0.0685 (16)	−0.0028 (14)	0.0087 (13)	0.0040 (14)
C22	0.0643 (18)	0.0710 (19)	0.088 (2)	−0.0119 (15)	0.0121 (15)	0.0219 (18)
C20	0.0856 (19)	0.079 (2)	0.0629 (17)	0.0042 (18)	0.0319 (14)	0.0024 (15)
C8	0.0588 (16)	0.083 (2)	0.0682 (17)	0.0069 (15)	0.0147 (13)	−0.0083 (16)
C19	0.0633 (16)	0.0639 (17)	0.0635 (16)	−0.0050 (14)	0.0205 (13)	0.0045 (14)
C21	0.0719 (19)	0.091 (2)	0.0750 (19)	0.0033 (18)	0.0346 (15)	0.0269 (18)

### Geometric parameters (Å, °)

**Table:**

O1—C5	1.374 (2)	C13—H13	0.93
O1—C2	1.376 (3)	C7—C8	1.383 (3)
N2—C1	1.286 (3)	C7—H7	0.93
N2—N1	1.356 (3)	C15—C14	1.368 (3)
C5—C4	1.353 (3)	C15—C16	1.374 (3)
C5—C6	1.446 (3)	C15—H15	0.93
C6—C11	1.385 (3)	C4—C3	1.409 (3)
C6—C7	1.389 (3)	C4—H4	0.93
C12—C17	1.383 (3)	C14—H14	0.93
C12—C13	1.396 (3)	C16—H16	0.93
C12—N1	1.405 (3)	C11—C10	1.380 (4)
N1—C18	1.439 (3)	C11—H11	0.93
N3—O3	1.215 (3)	C10—H10	0.93
N3—O2	1.229 (3)	C3—H3	0.93
N3—C9	1.473 (3)	C23—C22	1.380 (4)
C1—C2	1.432 (3)	C23—H23	0.93
C1—H1	0.93	C22—C21	1.373 (4)
C9—C10	1.366 (4)	C22—H22	0.93
C9—C8	1.368 (4)	C20—C21	1.374 (4)
C17—C16	1.385 (3)	C20—C19	1.377 (3)
C17—H17	0.93	C20—H20	0.93
C18—C23	1.370 (3)	C8—H8	0.93
C18—C19	1.374 (3)	C19—H19	0.93
C2—C3	1.351 (3)	C21—H21	0.93
C13—C14	1.370 (3)		
			
C5—O1—C2	107.12 (17)	C14—C15—H15	120.6
C1—N2—N1	120.19 (18)	C16—C15—H15	120.6
C4—C5—O1	109.13 (19)	C5—C4—C3	107.3 (2)
C4—C5—C6	135.0 (2)	C5—C4—H4	126.4
O1—C5—C6	115.92 (19)	C3—C4—H4	126.4
C11—C6—C7	117.9 (2)	C15—C14—C13	121.9 (2)
C11—C6—C5	121.4 (2)	C15—C14—H14	119.1
C7—C6—C5	120.7 (2)	C13—C14—H14	119.1
C17—C12—C13	118.7 (2)	C15—C16—C17	120.6 (2)
C17—C12—N1	120.5 (2)	C15—C16—H16	119.7
C13—C12—N1	120.8 (2)	C17—C16—H16	119.7
N2—N1—C12	116.83 (17)	C10—C11—C6	121.3 (3)
N2—N1—C18	121.64 (18)	C10—C11—H11	119.4
C12—N1—C18	121.41 (19)	C6—C11—H11	119.4
O3—N3—O2	123.9 (3)	C9—C10—C11	118.9 (3)
O3—N3—C9	118.1 (3)	C9—C10—H10	120.5
O2—N3—C9	118.0 (3)	C11—C10—H10	120.5
N2—C1—C2	119.7 (2)	C2—C3—C4	107.2 (2)
N2—C1—H1	120.1	C2—C3—H3	126.4
C2—C1—H1	120.1	C4—C3—H3	126.4
C10—C9—C8	121.9 (2)	C18—C23—C22	119.9 (3)
C10—C9—N3	118.9 (3)	C18—C23—H23	120
C8—C9—N3	119.1 (3)	C22—C23—H23	120
C12—C17—C16	120.4 (2)	C21—C22—C23	120.0 (3)
C12—C17—H17	119.8	C21—C22—H22	120
C16—C17—H17	119.8	C23—C22—H22	120
C23—C18—C19	120.1 (2)	C21—C20—C19	120.0 (3)
C23—C18—N1	120.3 (2)	C21—C20—H20	120
C19—C18—N1	119.6 (2)	C19—C20—H20	120
C3—C2—O1	109.2 (2)	C9—C8—C7	118.6 (3)
C3—C2—C1	133.3 (2)	C9—C8—H8	120.7
O1—C2—C1	117.4 (2)	C7—C8—H8	120.7
C14—C13—C12	119.7 (2)	C18—C19—C20	120.0 (3)
C14—C13—H13	120.2	C18—C19—H19	120
C12—C13—H13	120.2	C20—C19—H19	120
C8—C7—C6	121.4 (3)	C22—C21—C20	120.0 (3)
C8—C7—H7	119.3	C22—C21—H21	120
C6—C7—H7	119.3	C20—C21—H21	120
C14—C15—C16	118.8 (2)		
			
C2—O1—C5—C4	0.0 (2)	N1—C12—C13—C14	−179.9 (2)
C2—O1—C5—C6	179.54 (19)	C11—C6—C7—C8	0.4 (4)
C4—C5—C6—C11	−174.0 (3)	C5—C6—C7—C8	179.5 (2)
O1—C5—C6—C11	6.6 (3)	O1—C5—C4—C3	−0.2 (3)
C4—C5—C6—C7	7.0 (4)	C6—C5—C4—C3	−179.6 (3)
O1—C5—C6—C7	−172.4 (2)	C16—C15—C14—C13	1.2 (4)
C1—N2—N1—C12	179.0 (2)	C12—C13—C14—C15	−1.0 (4)
C1—N2—N1—C18	2.9 (3)	C14—C15—C16—C17	−0.6 (4)
C17—C12—N1—N2	−175.9 (2)	C12—C17—C16—C15	−0.2 (4)
C13—C12—N1—N2	4.2 (3)	C7—C6—C11—C10	−0.7 (4)
C17—C12—N1—C18	0.2 (3)	C5—C6—C11—C10	−179.8 (2)
C13—C12—N1—C18	−179.7 (2)	C8—C9—C10—C11	1.0 (4)
N1—N2—C1—C2	178.2 (2)	N3—C9—C10—C11	−179.1 (2)
O3—N3—C9—C10	177.7 (2)	C6—C11—C10—C9	0.0 (4)
O2—N3—C9—C10	−2.2 (4)	O1—C2—C3—C4	−0.3 (3)
O3—N3—C9—C8	−2.4 (4)	C1—C2—C3—C4	178.6 (3)
O2—N3—C9—C8	177.7 (2)	C5—C4—C3—C2	0.3 (3)
C13—C12—C17—C16	0.4 (4)	C19—C18—C23—C22	0.7 (4)
N1—C12—C17—C16	−179.5 (2)	N1—C18—C23—C22	179.5 (2)
N2—N1—C18—C23	−89.7 (3)	C18—C23—C22—C21	−0.1 (4)
C12—N1—C18—C23	94.4 (3)	C10—C9—C8—C7	−1.3 (4)
N2—N1—C18—C19	89.1 (3)	N3—C9—C8—C7	178.8 (2)
C12—N1—C18—C19	−86.8 (3)	C6—C7—C8—C9	0.6 (4)
C5—O1—C2—C3	0.2 (2)	C23—C18—C19—C20	−0.5 (4)
C5—O1—C2—C1	−178.92 (19)	N1—C18—C19—C20	−179.3 (2)
N2—C1—C2—C3	−176.1 (3)	C21—C20—C19—C18	−0.4 (4)
N2—C1—C2—O1	2.7 (3)	C23—C22—C21—C20	−0.8 (4)
C17—C12—C13—C14	0.2 (4)	C19—C20—C21—C22	1.0 (4)
